# Geographical variation in functional traits of leaves of *Caryopteris mongholica* and the role of climate

**DOI:** 10.1186/s12870-023-04410-9

**Published:** 2023-08-15

**Authors:** Xiao Yu, Ruoxuan Ji, Mingming Li, Xinli Xia, Weilun Yin, Chao Liu

**Affiliations:** National Engineering Research Center of Tree Breeding and Ecological Restoration, College of Biological Sciences and Technology, Beijing Forestry University, Beijing, 100083 China

**Keywords:** Leaf functional traits, Adaptation, Intra-specific variation, Climatic factors, *Caryopteris mongholica*

## Abstract

**Background:**

Quantifying intra-specific variation in leaf functional traits along environmental gradients is important for understanding species' responses to climate change. In this study, we assessed the degree of among and within populations variation in leaf functional traits and explored leaf response to geographic and climate change using *Caryopteris mongholica* as material, which has a wide range of distribution environments.

**Results:**

We selected 40 natural populations of *C. mongholica*, measured 8 leaf functional traits, analyzed the extent of trait variation among and within populations, and developed geographic and climatic models to explain trait variation between populations. Our results showed that the variation in leaf functional traits of *C. mongholica* was primarily lower within populations compared to among populations. Specifically, the leaf area (LA) exhibited higher variability both among and within populations, whereas leaf carbon content (LC) exhibited lower variation within populations but greater variation among populations. We observed a specific covariation pattern among traits and a strong linkage between morphological, economic, and mechanical traits. Increasing minimum temperature, precipitation of month, and seasonal precipitation differences all limited the growth and development of *C. mongholica*. However, it was observed that an increase in mean annual precipitation positively influenced the morphological development of its leaf.

**Conclusions:**

These results demonstrate the response of intra-specific trait variation to the environment and provide valuable insights into the adaptation of intra-specific leaf functional traits under changing climatic conditions.

**Supplementary Information:**

The online version contains supplementary material available at 10.1186/s12870-023-04410-9.

## Introduction

As global climate change accelerates, extreme weather events are becoming more common, posing a significant threat to plant survival and growth [1]. Leaf functional traits, such as leaf shape, specific leaf area, and leaf nitrogen content, exhibit a high level of sensitivity and responsiveness to environmental changes. Consequently, these traits can serve as reliable indicators of plant responses to climate change [2, 3]. Studies have shown that the survival strategies of plants and their capacity to utilize resources are closely linked to leaf functional traits [4, 5].

Plant leaf functional traits are subject to considerable variation in response to climate change and often exhibit some intra-specific variation [6, 7], resulting from a combination of genetic variation and phenotypic plasticity [8]. In addition, leaf traits show varying degrees of variation both within and among populations. For example, in Swiss subalpine grasslands, leaf nutrient concentrations vary significantly among populations [9]; European beech also shows substantial variation in leaf size, SLA, and Huber value among populations [10]; Warren's study revealed significant variation in leaf morphology and physiology of red ironwood eucalyptus among and within populations [11]; Similarly, Xu's research revealed significant differences in leaf functional traits among populations of *Cunninghamia lanceolata* [12]. Additionally, the majority of results indicating greater among populations variation than within populations variation at larger study scales [12–14].

The growth traits of plant exhibit significant variation due to the influence of environmental factors [15, 16]. The functional traits of leaves are sensitive to environmental factors, such as water, radiation, and temperature, which can be adjusted to achieve optimal utilization of limiting resources and enhance plant viability [17]. Leaf size and shape are considered morphological traits that play a crucial role in determining water use efficiency and the amount of light intercepted for photosynthesis [18, 19]; Mechanical traits, such as dry matter content (LDMC) and carbon content (LC), provide insights into a plant's resistance to physical injury [20]. Specific leaf area (SLA), nitrogen content (LN) and phosphorus content (LP) are considered economic traits that reflect a plant's utilization and adaptation to environmental factors, as well as nutrient stability and limitations [21]. For example, leaves under drought and low-temperature conditions often display smaller size, lower specific leaf area, and increased leaf nitrogen content, which can improve water utilization and enhance photosynthetic efficiency [22–24].

However, the relationship between leaf traits and environmental factors is not fixed and can vary depending on the scale of the study. Furthermore, when environmental changes occur, plant traits often undergo simultaneous changes, rather than singularly, resulting in complex synergistic relationships among plant functional traits that collectively regulate and sustain plant life activities. For instance, in arid regions characterized by low precipitation and high evapotranspiration rates, woody plants have adapted to drought conditions by reducing transpiration through smaller leaf areas and higher tissue density compared to plants in other habitats at the same latitude [25]. However, studies have also indicated that in arid regions, the connection between plant leaf traits and environmental variables tends to diminish [26]. Consequently, the relationship between traits and environmental variables may not be clearly defined at different scales of study in arid and semi-arid regions. Understanding the drivers of plant trait variation and the interrelationships among traits in this region is crucial for accurately characterizing plant functional diversity in global carbon-climate models.

The current research on plant functional traits has predominantly focused on comparing the average trait values among species at large scales [27], which has overlooked the importance of intra-specific trait variation in maintaining species coexistence and community dynamics. There is growing evidence that intraspecific trait variation has a significant and non-negligible impact on species identity and ecosystem function due to phenotypic plasticity and local adaptation. Therefore, to advance our understanding of environmental effects on trait variation and predict species responses to climate change, it is essential to quantify intra-specific trait variation along environmental gradients. This is particularly important for widely distributed plants [28–30].

*Caryopteris mongholica,* a shrub species classified under the genus *Caryopteris* in the family *Labiatae*, is currently endangered. It primarily occupies arid and semi-arid regions [31] and plays a significant role in sand fixation, soil conservation, the retardation of desertification, and the stabilization of native ecological environments. Being the northernmost species within the *Caryopteris* genus, it holds a critical phylogenetic position [32]. Moreover, its population distribution encompasses a wide range of environmental conditions and exhibits substantial variation [33]. In response to anticipated future climate changes, its distribution area is gradually shifting northward [34]. Consequently, it serves as a valuable resource for studying intra-specific leaf trait variation within the species. However, the existing studies on *C. mongholica* are predominantly confined to limited geographical areas, and there is a lack of comprehensive comparisons of functional traits on broader scales. This limitation restricts our depth of understanding regarding phenotypic differentiation and ecological adaptation in this species. Therefore, in this study, we used *C. mongholica* as material to assess the degree of variation among populations and within populations in leaf functional traits across its range and explore leaf responses to geographic and climate change. We sampled *C.mongholica* leaves from various regions during July–September 2018–2021 and made the following hypotheses: (1) The functional traits of leaves in *C.mongholica* exhibit varying degrees of variation both within and among populations within its distribution range, and there are covariation patterns among these traits. (2) Each functional trait of leaf within the species demonstrates a response to changes in geographic gradients. (3) Each functional trait of leaf within the species adjusts its survival strategy in response to changes in climate. Testing these hypotheses will enhance our understanding of how environmental factors influence plant ecological strategies.

## Materials and methods

### Study area

**Fig. 1:**
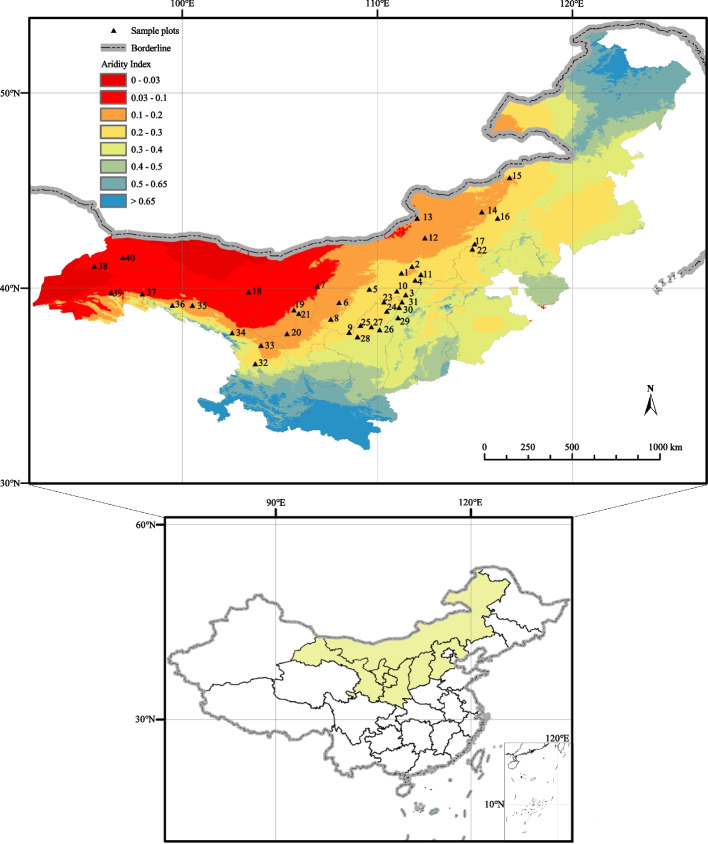
Distribution of sample sites of *C. mongholica* communities in the study area. Aridity Index: < 0.03: Hyper arid, 0.03—0.2: Arid, 0.2—0.5: Semi-Arid, 0.5—0.65: Dry sub-humid, > 0.65: Humid. (The maps are created by authors using the ArcGIS software)

We selected 40 representative populations of *C. mongholica* (Fig. 1) based on the natural population distribution provided by the China National Specimen Resources Sharing Platform (http://www.nsii.org.cn/2017/home.php), These populations were located at least 25 km apart and covered the primary natural geographical distribution area of *C. mongholica* in northern China. The 40 sample sites were mainly located in northwestern China (36°06′N—45°39′N and 96°21′E—116°45′E), which experiences a typical temperate continental arid climate with relatively low precipitation (annual mean precipitation 67—475 mm), strong temperature heterogeneity (annual mean temperature 1.03—9.59 °C), and a large altitude span (911—2805 m). The specific locations of the 40 sample sites are shown in Table S1.

### Sample collection and determination

Between July and August of each year from 2018 to 2021, we established three 10 m × 10 m survey sample squares in each survey area with a spacing of at least 50 m between each square. Within each square, We selected ten healthy plants and collected 30–50 mature and morphologically intact leaves from each plant. The leaves were then scanned using a flatbed scanner (CanonScan LiDE 120, Canon, RoHS, WEEE), and the scanned images were analyzed using Image-Pro Plus 6.0 (Olympus, Tokyo, Japan) to obtain leaf morphological parameters. After weighing, the leaves were dried at 75 °C to a constant weight, and we calculated the specific leaf area of each sample from the corresponding leaf weight and area. Next, we ground the leaves and passed them through a 100-mesh sieve to determine their carbon, nitrogen, and phosphorus content. The carbon and nitrogen content of the samples were measured using a combined C/N analyzer (2400II CHNS/O, Element Analyzer, Perkin-Elmer, Boston, MA, United States), the determination of the phosphorus content was carried out using a molybdenum-antimony anti-colorimetric method [35].

Finally, we obtained a total of 8 trait indices, including leaf area (LA), perimeter (LPE), aspect ratio (LRA), specific leaf area (SLA), dry matter content (LDMC), carbon (LC), nitrogen (LN) and phosphorus (LP) content per unit mass, for 40 natural populations of *C. mongholica*.

$$\begin{array}{c}\mathrm{LRA }=\mathrm{\,Leaf\,length\,}/\mathrm{\,Leaf\,width}\\ \mathrm{LDMC\,}=\mathrm{\,Leaf\,dry\,weight\,}/\mathrm{\,leaf\,fresh\,weight}\\ \mathrm{SLA }=\mathrm{\,Leaf\,area\,}/\mathrm{\,LDMC}\end{array}$$

### Data sources and processing

#### Degree of variation and covariation in leaf functional traits

The variability of leaf traits within and among 40 populations of *C. mongholica* was investigated using SPSS 17.0 (SPSS Inc, Chicago, USA). The coefficient of variation (CV) for each of the 8 leaf traits within each population was calculated and averaged across all populations to assess the degree of trait variation within populations. For trait variation among populations, the degree of variation was quantified using two metrics: Phenotypic dissimilarity (PhD) index [36] and multiplicity of variation. In addition, we defined the leaf trait spaces using Principal Component Analysis (PCA) on imputed trait data by using the funspace function in the ‘funspace’ R package [37], resulting in the extraction of two trait principal components (T-PC1 and T-PC2). Furthermore, correlation analysis was performed to examine the relationships between the traits (corresponding to the first question).

#### Relationship between leaf traits and geographical gradients (longitude, latitude, altitude)

Meteorological data were acquired from WorldClim (http://www.worldclim.org/version2) with a spatial resolution of 30 s, and bioclimatic data for the sample plots were obtained through ArcGIS 10.6 software. A total of 17 climate variables were extracted, but after removing highly correlated variables (*r* > 0.90; Table S2), eight climate factors remained. Eight factors included five temperature variables and three precipitation variables (Table S3). Principal component analysis was used to reduce the dimensions of the data, resulting in the extraction of two principal components (E-PC1 and E-PC2), which accounted for 73.49% of the total variance. E-PC1 explained 49.24% of the total variance, the minimum temperature of the coldest month and mean temperature of the driest quarter had higher loadings on E-PC1. E-PC2 explained 24.25% of the total variance, and the mean annual precipitation had higher loadings on E-PC2. The correlation analysis revealed that E-PC1 was significantly correlated with longitude, latitude, and altitude, while E-PC2 was significantly correlated with longitude and altitude (*p* < 0.01, Table S3).

The effects of longitude, latitude, and altitude on the two principal component axes (T-PC1 and T-PC2) of the trait were analyzed using linear mixed model from the lme4 package of R 3.6.2, sites were treated as random effect (corresponding to the second question). After controlling for the effects of the two climatic principal components, we performed partial correlation analysis to identify the unique associations between geographic variables and leaf traits. For the visualization and prediction of each leaf functional trait, Kriging interpolation was utilized, and the data were examined to exclude the corresponding second-order trends and transformations. The predictions were made for the provinces where *C. mongholica* was distributed in the study area, this prediction process was carried out using ArcGIS 10.6.

#### Relationship between leaf traits and climate

Linear mixed model from the lme4 package was used to examine the relationship between leaf traits and climate variables (corresponding to the third question). Two sets of models were implemented: one considering the first two principal components (E-PC1 and E-PC2) of climate variables, and the other including all eight climate variables. The analysis was performed in R version 3.6.2.

### Method statement

We ensured that we have permission to collect *C. mongholica*, the plant collection and investigation was approved by the Academy of Forestry Science, the Inner Mongolia Autonomous Region, China. The samples were carefully identified by Professor Meng Ji of Academy of Forestry Science and Professor Weilun Yin at Beijing Forestry University based on the descriptions in Flora of China, a voucher specimen was deposited in the Herbarium of Plant Biology Department, Beijing Forestry University with an accession number BJFU-CM117.

## Results

### Degree of variation in leaf functional traits

Leaf traits exhibited varying levels of among and within populations variability across the 40 sample areas. The within populations variability of 8 leaf functional traits in the wild population of *C. mongholica* was generally lower than the among populations variability. Within populations variation coefficients for leaf traits ranged from 4.28% to 17.53%, while among populations PhD index ranged from 9.21% to 22.54%. The largest variations were observed within and among populations for LA. On the other hand, LC exhibited minimal variation within populations but greater variation among populations. LRA showed less variation both within and among populations. All traits exhibited a variance variation of more than onefold between populations, and there were significant differences in leaf functional traits among populations (Table 1).

**Table 1:** Leaf trait variation of *C. mongholica* from 40 sites across its geographic distribution

Leaf Trait	Range	Mean	Std. Deviation	Std. Error	Fold range	PhD (among populations)	CV (within populations)
LPE (mm)	49.31 ~ 107.12	75.34	16.35	2.58	2.17	18.95%	10.24
LA (mm^2^)	67.46 ~ 295.05	158.16	59.94	9.48	4.37	22.54%	17.53
LRA	3.60 ~ 7.19	4.96	0.68	0.11	2.00	9.21%	8.90
SLA(cm^2^/g)	66.69 ~ 161.94	110.83	20.80	3.29	2.43	14.82%	7.17
LN(g/kg)	18.28 ~ 32.95	25.22	3.62	0.57	1.80	15.15%	8.60
LP(g/kg)	0.77 ~ 4.34	1.88	0.68	0.11	5.61	13.55%	13.97
LC(g/kg)	324.59 ~ 470.47	416.04	39.30	6.21	1.45	18.28%	4.28
LDMC(g/kg)	205.98 ~ 695.46	330.67	109.82	17.36	3.38	17.02%	6.78

**Fig. 2:**
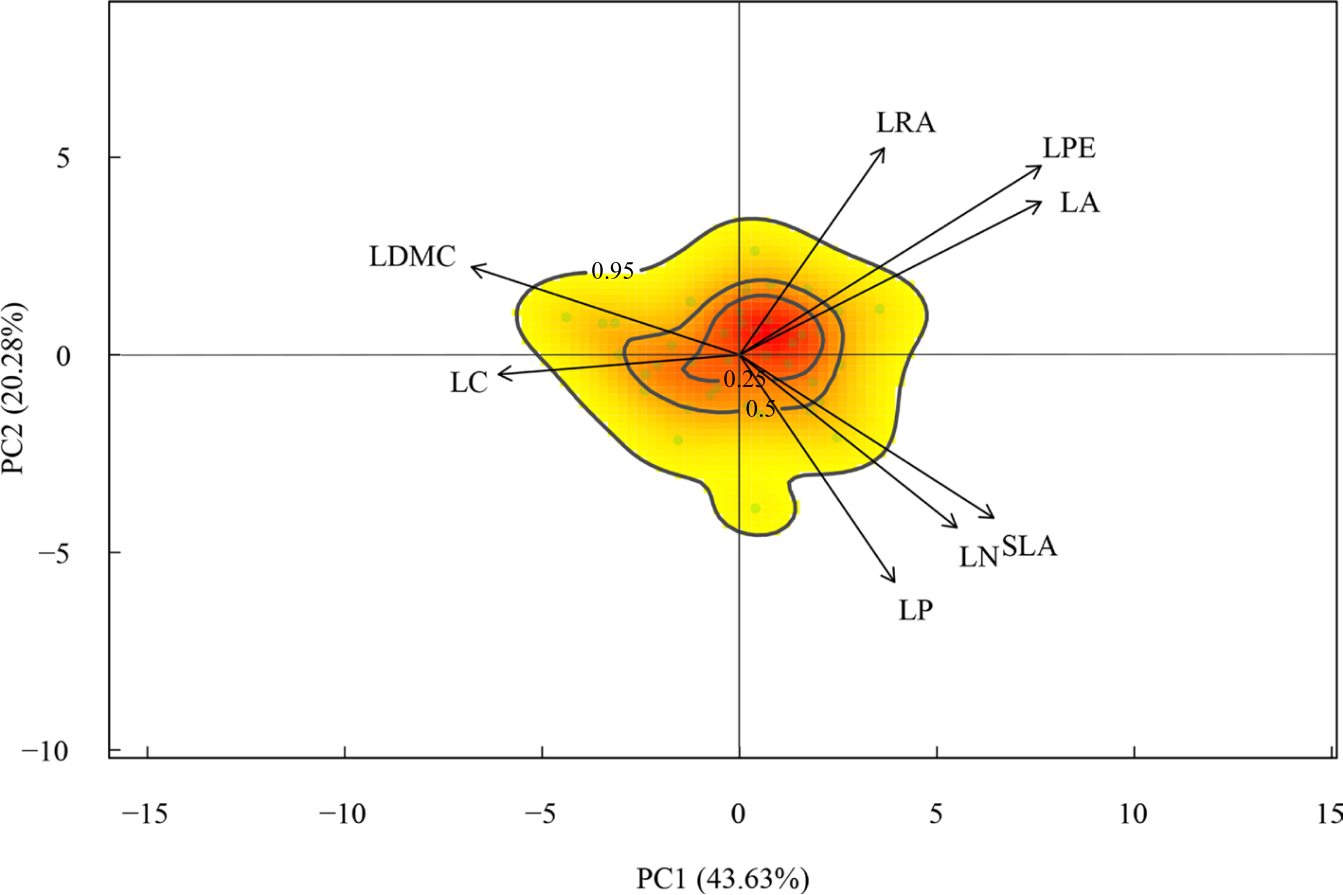
Towards a leaf trait space. The trait space is defined by a PCA on leaf trait data. Colors indicate the probabilistic distribution of trait combinations in the functional trait space defined by a PCA (red = high probability; yellow = low probability). Contour lines indicate 0.95, 0.50, and 0.25 quantiles of the probability distribution. The output shows that there one hotspot. The variance explained by each component and the loadings of the original traits are also shown. LL, LPE, LRA (morphological traits); SLA, LN, LP (economic traits); LDMC, LC (mechanical traits)

PCA analysis of leaf functional traits revealed that the eight traits could be categorized into two dimensions (T-PC1 and T-PC2). The combined contribution of these two principal component axes accounted for 63.91% of the total variance. Among the traits, LPE, LA, SLA, and LDMC exhibited larger vector lengths on the T-PC1 axis, while LRA and LP showed larger vector lengths on T-PC2. Additionally, traits exhibited a discernible covariance pattern across the three aspects of morphology, economy, and mechanical (Fig. 2).

Correlation analysis (Fig. S1) confirmed significant positive correlations (*p* < 0.01) between leaf morphological traits (LPE, LA, LRA) and negative correlations (*p* < 0.01) between morphological traits and mechanical traits (LDMC, LC). Additionally, significant positive correlations (*p* < 0.01) were observed among economic traits (SLA, LN, LP). Furthermore, SLA exhibited significant positive correlations (*p* < 0.05) with morphological traits and negative correlations (*p* < 0.05) with mechanical traits. However, there were no significant correlations between LRA, mechanical traits, and economic traits. In summary, the analysis showed that *C. mongholica* exhibited general correlations among leaf traits, with close relationships among morphological, mechanical, and economic traits.

### Effect of geography on leaf traits

The linear mixed model analysis revealed a significant correlation between the T-PC1 axis of the traits and longitude (*p* < 0.05), while latitude and altitude did not show significant correlations with the two PC axes of the trait (Table 2). Regression analysis further confirmed a significant correlation between longitude and T-PC1 axis (Fig. S2). The results of the partial correlation analysis revealed that latitude showed a significant correlation with LC only, while longitude exhibited significant correlation with LPE, LA, LDMC, LC, and SLA. Additionally, altitude showed a significant correlation with LDMC under uncontrolled climate conditions (Table 3). Both the linear mixed models and the partial correlation analysis consistently indicated that leaf traits of *C. mongholica* were primarily influenced by longitude.

**Table 2:** T-PC1 and T-PC2 of leaf traits in relation to longitude/latitude/altitude

Varible	Fixed effects		
	Predictor	Estimate	*p* value
T-PC1	Latitude	-11.29	0.60
	Longitude	-25.09	0.03*
	Altitude	0.03	0.85
T-PC2	Latitude	3.66	0.59
	Longitude	-1.63	0.65
	Altitude	0.01	0.74

* means *p* < 0.05

**Table 3:** Correlation coefficients between leaf traits and latitude, longitude and bioclimatic principal components (E-PC1 and E-PC2)

	LPE	LA	LRA	LDMC	LC	SLA	LN	LP
Latitude	0.114	0.280	-0.188	-0.181	-0.420**	0.234	-0.013	0.039
Latitude (Control E-PC1)	-0.131	-0.030	-0.314	0.152	-0.309	-0.228	-0.044	0.074
Latitude (Control E-PC2)	0.152	0.308	-0.151	-0.302	-0.452**	0.299	-0.002	0.040
Latitude (Control E-PC1 and E-PC2)	-0.066	0.019	-0.237	-0.011	-0.381*	-0.139	-0.022	0.077
Longitude	0.341*	0.397*	0.140	-0.522**	-0.378*	0.386*	0.177	0.109
Longitude (Control E-PC1)	0.284	0.274	0.196	-0.457**	-0.258	0.234	0.205	0.126
Longitude (Control E-PC2)	0.252	0.405**	-0.157	-0.250	-0.371*	0.230	0.169	0.152
Longitude (Control E-PC1 and E-PC2)	0.154	0.245	-0.182	0.001	-0.204	-0.118	0.246	0.225
Altitude	-0.237	-0.232	-0.177	0.435**	0.309	-0.191	-0.128	-0.063
Altitude (Control E-PC1)	-0.227	-0.220	-0.181	0.433**	0.302	-0.177	-0.127	-0.062
Altitude (Control E-PC2)	-0.075	-0.183	0.217	-0.01	0.311	0.157	-0.109	-0.112
Altitude (Control E-PC1 and E-PC2)	-0.050	-0.149	0.213	-0.057	0.286	0.23	-0.109	-0.112

** means *p* < 0.01, * means *p* < 0.05

**Fig. 3:**
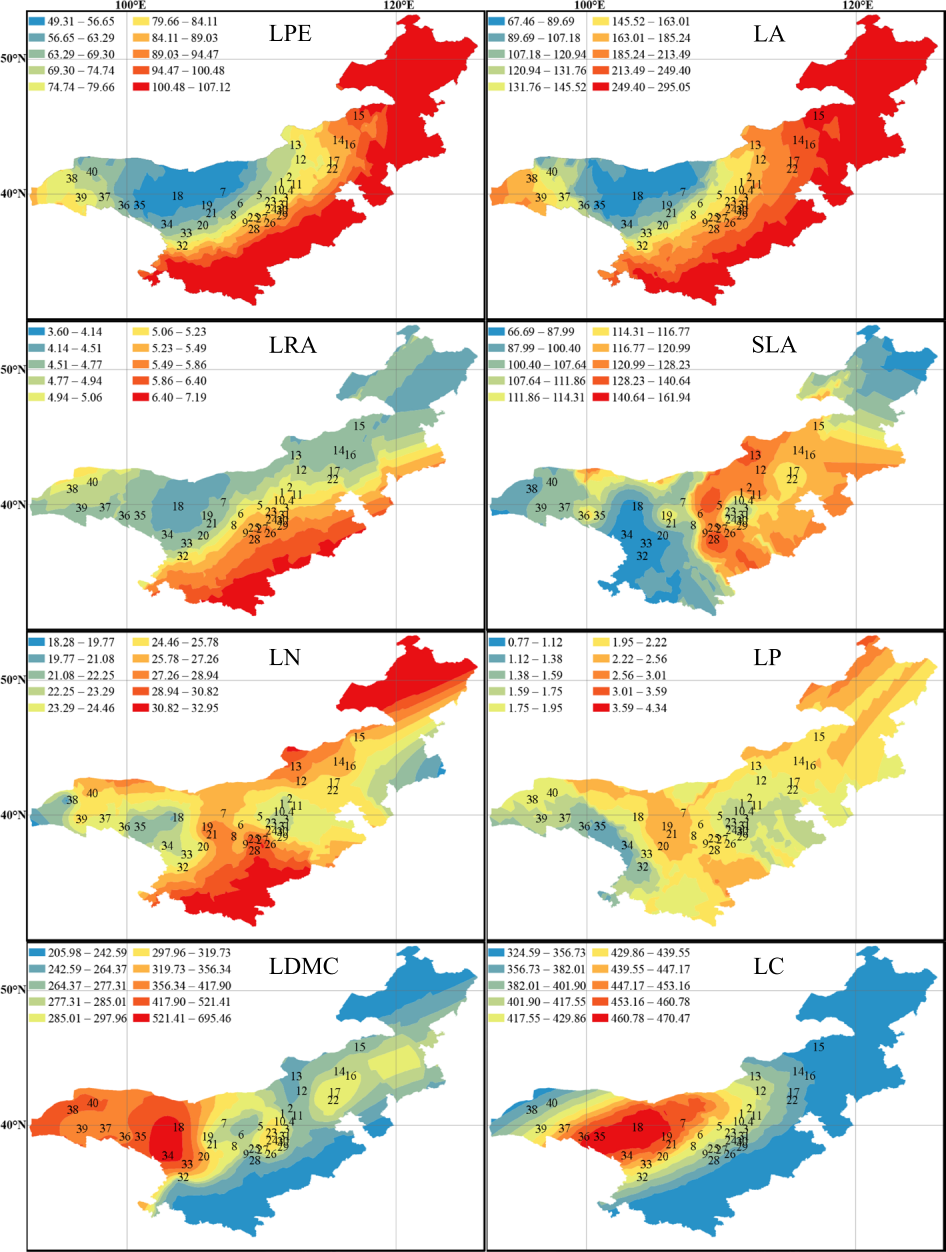
Prediction of leaf trait pattern distribution. (The maps are created by authors using the ArcGIS software)

The distribution patterns of leaf traits were predicted and visualized using interpolation techniques (Fig. 3). The predictions indicated that the morphological traits (LPE and LA) of leaves exhibited a similar trend, gradually decreasing from southeast to northwest. Moreover, the LRA demonstrated a gradual increase from south to north within the range of 95°E-120°E, suggesting a pronounced bias towards ovoid growth in leaves located further south in this range. Furthermore, the SLA of *C. mongholica* leaves located east of 107°E was significantly higher than that of leaves located west of 107°E. LN and LP displayed a distribution pattern characterized by higher values in the central region and lower values on both sides, delineated by sample site 19. Similarly, LDMC was also divided by 107°E, with higher LDMC in the west compared to the east. West of 107°E, LDMC was centered around sample site 18 (39.79°N, 103.41°E) and gradually decreased in all directions. LC was centered around sample site 18 and showed a gradual decrease in all directions.

After controlling for hydrothermal conditions (E-PC1 and E-PC2), the significant correlations between leaf traits and latitude and longitude disappeared (Table 3). Regarding the relationship between LC-latitude, the significant correlation disappeared when controlling the influence of E-PC1. However, significant correlation remained when controlling the influence of E-PC2 (Table 3). Therefore, the patterns of LC along the latitudinal gradient were more influenced by temperature (represented by E-PC1) than precipitation (represented by E-PC2). Regarding the trait-longitude relationship, significant correlation with LDMC remained when controlling the influence of E-PC1, while significant correlations with LPE, LA, SLA, and LC disappeared. When controlling the influence of E-PC2, significant correlations with LA and LC still existed, but significant correlations with LPE, LDMC, and SLA disappeared. All significant correlations disappeared when controlling E-PC1 and E-PC2 simultaneously (Table 3). Thus, regarding the seven leaf traits along the longitude gradient, LA, and LC were more influenced by temperature (represented by E-PC1), while LDMC was more influenced by precipitation (represented by E-PC2), LPE and SLA were influenced by both climate variables (represented by E-PC1 and E-PC2). Regarding the altitude-trait relationship, only LDMC was significantly influenced by altitude, and along the altitude gradient, LDMC was more influenced by the climatic variables represented by E-PC2 than E-PC1 (Table 2).

### Effect of climate on leaf traits

The results obtained from the linear mixed model analysis revealed important relationships between leaf traits and climate variables. Specifically, E-PC1 exhibited a significant negative correlation with LA and a significant positive correlation with LC. Similarly, E-PC2 exhibited a significant negative correlation with LDMC, while displaying significant positive correlations with LPE, LRA, and SLA (Table 4A). Temperature and precipitation were found to exert substantial effects on leaf traits (Table 4B). Among the eight climate factors, five were found to be the strongest predictors of leaf trait variation (Table 4B). These influential climate factors include temperature seasonality (BIO2), minimum temperature of coldest month (BIO4), annual precipitation (BIO10), precipitation of driest month (BIO12), and precipitation seasonality (BIO13). Specifically, BIO2 demonstrated a significant negative effect on LRA and LC, while the BIO4 displayed a significant negative effect on LPE and LRA, and a positive effect on LDMC. Moreover, annual precipitation (BIO10) exhibited a significant positive effect on LPE and a significant negative effect on LC. Additionally, precipitation of driest month (BIO12) exerted a significant negative effect on LPE and LA, but a significant positive effect on LC. Furthermore, the precipitation seasonality (BIO13) was found to have a significant negative effect on LPE and LRA, while showing a significant positive effect on LC. It is worth noting that SLA and LDMC were significantly influenced by E-PC2, representing precipitation, although the impact of a single climate factor was not significant.

**Table 4:** Results of linear mixed model of *C. mongholica* leaf traits with climate variables

(A) principal components of climate variables
LPE = 19.46–0.02PC1 + 0.06PC2*
LA = -105.88–0.17PC1* + 0.14PC2
LRA = 4.46 + 0.001PC1 + 0.003PC2*
LDMC = 862.34 + 0.21PC1-0.51PC2**
LC = 611.86 + 0.14PC1*-0.09PC2
SLA = 28.25–0.04PC1 + 0.07PC2*
LN = 21.12–0.001PC1 + 0.004PC2
LP = 1.42–0.0007PC1-0.0003PC2
(B) climate variables
LPE = 89.30 + 20.21BIO1 + 0.06BIO2-11.24BIO3-12.61BIO4* + 1.14BIO7 + 0.09BIO10*-8.84BIO12*-1.17BIO13*
LA = 174.87 + 59.57BIO1 + 0.45BIO2-41.45BIO3-34.86BIO4 + 8.21BIO7 + 0.29BIO10-33.86BIO12*-3.35BIO13
LRA = 4.33 + 1.40BIO1**-0.01BIO2*-0.47BIO3-0.94BIO4***-0.11BIO7 + 0.001BIO10-0.15BIO12-0.04BIO13*
LDMC = 1022.26–44.28BIO1-0.10BIO2 + 7.42BIO3 + 31.60BIO4 + 0.98BIO7-0.13BIO10-42.06BIO12 + 1.84BIO13
LC = 539.06 + 17.86BIO1-0.36BIO2*-5.86BIO3-1.12BIO4-9.67BIO7*-0.34BIO10*** + 15.16BIO12* + 3.17BIO13**
SLA = 23.41 + 8.12BIO1-0.07BIO2-1.14BIO3-7.25BIO4-1.77BIO7 + 0.01BIO10 + 6.28BIO12-0.07BIO13
LN = 36.94 + 0.86BIO1 + 0.01BIO2-0.36BIO3-0.22BIO4-0.40BIO7 + 0.01BIO10-0.67BIO12-0.22BIO13
LP = 4.31 + 0.51BIO1 + 0.003BIO2-0.28BIO3-0.16BIO4-0.05BIO7-0.002BIO10 + 0.10BIO12-0.04 BIO13

* in the equation indicates that the parameter is significant. *** means *p* < 0.001, ** means *p* < 0.01, *means *p* < 0.05

## Discussion

The 8 functional traits of *C. mongholica* leaves showed varying degrees of intra-specific variation both among and within populations, with within populations coefficients of variation ranging from 4.28%-17.53% and among populations coefficients of variation ranging from 9.21%-22.54% (Table 1). The variation was greater among populations than within population (Table 1). Microhabitat and genetic effects typically influence trait variation within populations [38], while geographic and climatic factors influence trait variation among populations [39–41]. In our study, *C. mongholica* leaf traits may be more influenced by geography and climate.

In this study, both within and among populations, leaf area exhibited the highest degree of variation. Under natural conditions, leaf size is a crucial trait that determines plant water use efficiency and photosynthetic efficiency [19, 42]. Furthermore, leaf size is highly responsive to environmental changes and possesses greater phenotypic plasticity [43]. Second, the LP was also more variable within and among populations. The LP of *C. mongholica* in this study was slightly higher than the national average for terrestrial plants (1.46 g/kg) [44]. It has been confirmed that the soil phosphorus content in most parts of China is lower than the global average, resulting in an overall low LP in plants [44], indicating that plant growth is more likely to be phosphorus-limited. Additionally, the variability of soil phosphorus content in China is increasing from the humid zone to the arid/semi-arid zone with a large overall variation [45], which may also explain the high variability of plant leaf LP observed in this study. In contrast, LC exhibited lower variation within populations but greater variation among populations. This may be attributed to the fact that carbon is a skeletal element that constitutes the plant, providing stability and support. As a result, LC within a given region tends to remain relatively consistent [46]. However, differences in environmental conditions, such as climate and soil, between populations can lead to variations in carbon content among populations.

Functional traits are not independent of each other and are naturally selected to form an optimal combination of traits that can adapt to specific environments [47, 48]. In this study (Fig. 2), we found significant correlations between morphological traits (LPE, LA, LRA) of *C. mongholica* leaves were significantly positively correlated with each other. Meanwhile, two mechanical traits (LDMC, LC) were significantly and negatively correlated with morphological traits. In addition, we found significant positive correlations between specific SLA, LN, and LP, positive correlation between SLA and morphological traits, as well as the negative correlation between SLA and mechanical traits (LDMC, LC), is in line with the global pattern of plant leaf trait correlations [49]. However, correlations between LN and LP with morphological and mechanical traits were less significant. Overall, there is a specific pattern of covariation among leaf traits in *C. mongholica* that adapts to the more heterogeneous northwestern arid habitat by adopting different trait combinations, with distributional trade-offs between structural toughness and rapid growth.

In this study, it was observed that almost all leaf traits of *C. mongholica* exhibited variation with geographic and climatic variables. Although the LN and LP were not significantly correlated with geographic and climatic factors, they were significantly correlated with SLA, which itself was influenced by climatic factors (Table 4). Therefore, it can be inferred that climatic factors indirectly influence leaf LN and LP by affecting SLA.

On the spatial gradient (Fig. 3), the morphological traits (LPE, LA) of *C. mongholica* leaves exhibit a gradual decrease from southeast to northwest of China. This trend is consistent with the variation observed in the average leaf size of woody plants in China, as reported by Li et al. [50]. Specifically, in warm and humid regions, plants tend to have larger leaves, while in arid and cold regions, plants typically exhibit smaller leaves. However, LRA demonstrated a tendency to become progressively larger with latitude from north to south. Plants in response to the environment with increased precipitation, by changing leaf shape (Fig. 3) and becoming progressively more rounded to facilitate water and air exchange with the outside world [51]. Both SLA and LDMC displayed different trends on both sides of 107°E, which suggests that factors beyond precipitation and temperature might be influencing these variations. In close proximity to the 107°E longitude lie the north–south Helan Mountains and the north–south flowing bend of the Yellow River. The presence of these mountains and the distribution of Yellow River's water resources may explain the differences in leaf traits between the two sides. The pattern of leaf traits along the spatial gradient is primarily influenced by climate (Table 3). The relationship between climate and leaf traits is complex, and changes in leaf functional traits cannot be predicted by a single climate factor (Table 3).

The outcomes derived from the analysis conducted using a linear mixed model (Table 4B) demonstrated that certain climatic variables, namely the BIO4, BIO12, and BIO13, exhibited a negative impact on leaf size (LPE, LA). Conversely, an increase in annual precipitation (BIO10) was found to positively contribute to the development of leaves. When exposed to extremely cold and dry conditions, *C. mongholica* produced smaller leaves due to the increased minimum temperature, increased precipitation of driest month, and increased seasonal variation in precipitation. The coldest month (January) and driest month (December) are both in winter, and deciduous plants adjust their metabolism to adapt to the winter season, which affects leaf development in the next growing season. Li et al. [52] employed Maxent software to forecast the potential habitat of *C. mongholica* in China. The study's results demonstrated that *C. mongholica* exhibited a low water requirement for its growth. We used Maxent to predict the distribution of *C. mongholica* in the fitness zone. The results showed that the survival of *C. mongholica* was severely limited in the coldest months when the minimum temperature was greater than -18 °C and the minimum rainfall was greater than 2 mm (Fig. S3). *C. mongholica* exhibits some cold tolerance and fear of flooding during winter to adapt to the dry and cold environment.

In this study, we observed that LDMC and LC, although displaying covariation, do not exhibit identical responses to geographical and climatic variables. LDMC is primarily influenced by longitude, while LC is mainly influenced by latitude. Among the climatic variables examined, LDMC exhibited a significant negative correlation with E-PC2, which primarily represents precipitation. On the other hand, LC displayed a significant positive association with E-PC1, representing temperature. Additionally, LC was found to be significantly influenced by multiple individual climatic factors. Consequently, it is plausible to speculate that although LDMC and LC exhibit a covariance pattern in trait variation, this pattern could be attributed to variations in other traits. Additionally, it should be noted that other local environmental factors that were not considered in this study, such as soil fertility, irradiation, and species richness, could also influence plant traits significantly. These factors have been significantly correlated with leaf traits in previous studies [53–55]. Currently, there is a debate regarding the relative strength of climate-dominant factors, such as temperature and precipitation, on leaf trait variation [56, 57]. Nevertheless, this study highlights that the variation in leaf functional traits within populations is driven by a combination of temperature and precipitation.

## Conclusions

The functional traits of *C.mongholica* leaves exhibit varying degrees of trait variation within and among populations. In general, the within population variation tends to be smaller compared to the among population variation, and specific pattern of covariation among traits. Variation in most leaf traits is influenced by climatic conditions along a geographic gradient, with longitude having a stronger influence than latitude and altitude on leaf trait variation. Temperature and precipitation, in combination, significantly impact leaf trait variation, with temperature seasonality (BIO2), minimum temperature of coldest month (BIO4), annual precipitation (BIO10), precipitation of driest month (BIO12), and precipitation seasonality (BIO13) having a greater impact. Increasing minimum temperature, precipitation in the driest month, and seasonal variation in precipitation limit the growth and development of *C. mongholica*. Moreover, morphological traits, mechanical traits, and economic traits are intricately interconnected and regulate the trait development of leaves.

### Supplementary Information

**Additional file 1: Fig. S1.** Correlation coefficients of plant leaf functional traits in *C. mongholica*. *** means *p*<0.001, ** means *p*<0.01, * means *p*<0.05, means *p*<0.1.

**Additional file 2: Fig. S2.** The effects of longitude, latitude, and altitude on the two principal component axes (T-PC1 and T-PC2) of the trait. Note: The solid line is significant and the dashed line is insignificant.

**Additional file 3: Fig. S3.** Response curves between the probability of presence and climate variables of *C. mongholica*. The y-axis is the probability of existence of C. *mongholica*. Blue: mean±one standard deviation.

**Additional file 4: Table S1.** Site characteristics for 40 sites of *C. mongholica* communities across its distribution in China.

**Additional file 5: Table S2.** Bivariate relationships among climatic variables.

**Additional file 6: Table S3.** Principal components analysis for climatic variables estimated for provenances (40 sites) of *C. mongholica*.

## Data Availability

The plant materials were collected from natural population in geographic distribution of *C. mongholica*. The datasets generated during the current study has been deposited in the Science Data Bank repository (https://www.scidb.cn). Data access link: https://cstr.cn/31253.11.sciencedb.07946. https://doi.org/10.57760/sciencedb.07946. All data generated during the current study are included in this published article.

## Abbreviations

LA: Leaf area
LPE: Leaf perimeter
LRA: Leaf aspect ratio
SLA: Specific leaf area
LDMC: Leaf dry matter content
LC: Leaf carbon content
LN: Leaf nitrogen content
LP: Leaf phosphorus content
CV: Coefficient of variation
PhD: Phenotypic dissimilarity
PCA: Principal component analysis
BIO1: Annual Mean Temperature
BIO2: Temperature Seasonality (standard deviation *100)
BIO3: Maximum Temperature of Warmest Month
BIO4: Minimum Temperature of Coldest Month
BIO7: Mean Temperature of Driest Quarter
BIO10: Annual Precipitation
BIO12: Precipitation of Driest Month
BIO13: Precipitation Seasonality (Coefficient of Variation)
