# Supplementary material for: Geographical variation in functional traits of leaves of Caryopteris mongholica and the role of climate
Source: BMC Plant Biol. 2023 Aug 15;23:394. doi: 10.1186/s12870-023-04410-9 (PMC10426221; doi:10.1186/s12870-023-04410-9)
Supplement: Supplementary file 1 — Additional file 1: Fig. S1. Correlation coefficients of plant leaf functional traits in C. mongholica. *** means p<0.001, ** means p<0.01, * means p<0.05, means p<0.1. [file 12870_2023_4410_MOESM1_ESM.docx]

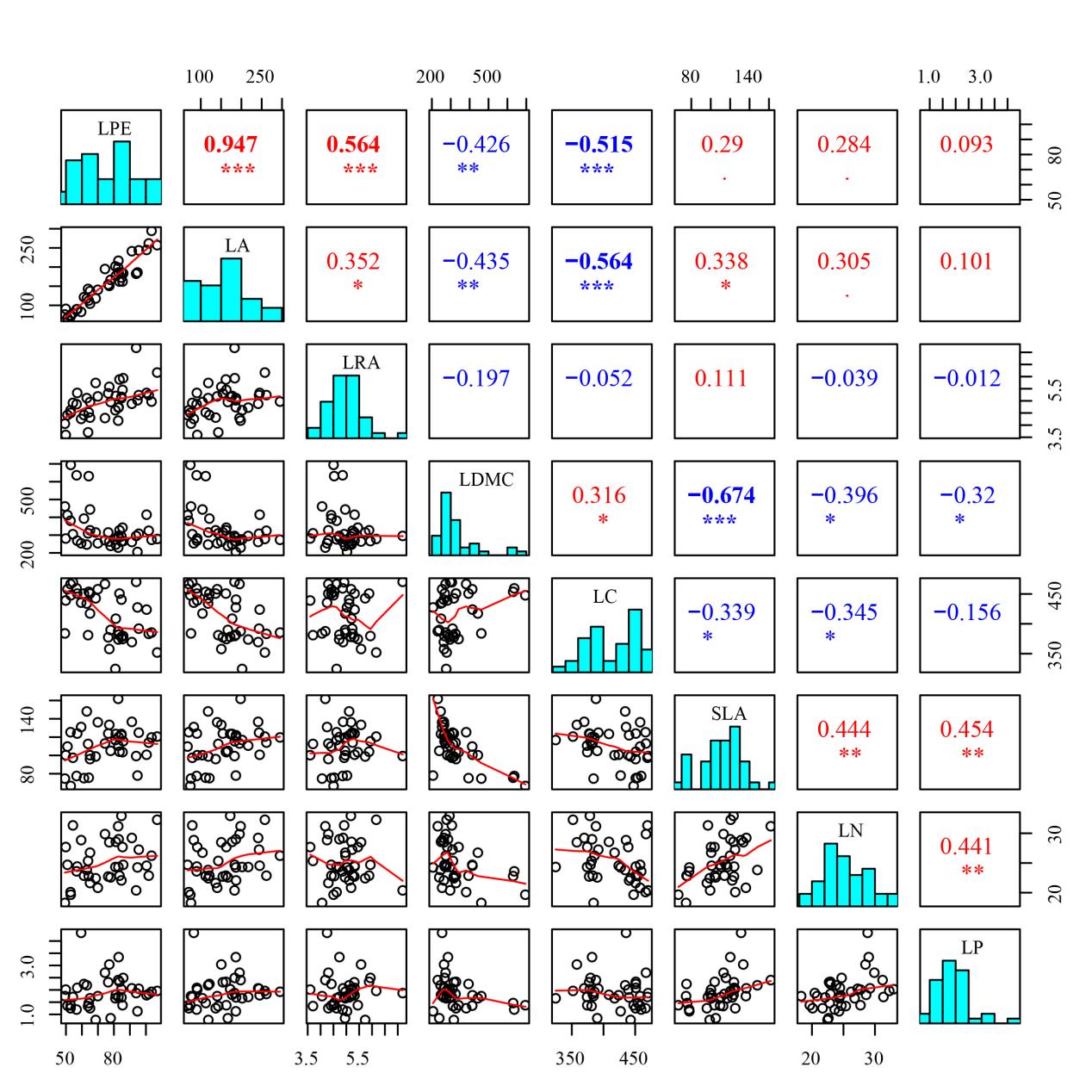


Fig.S1. Correlation coefficients of plant leaf functional traits in *C. mongholica.* *** means p<0.001, ** means p<0.01, * means p<0.05, ^.^ means p<0.1.
