# Supplementary material for: Geographical variation in functional traits of leaves of Caryopteris mongholica and the role of climate
Source: BMC Plant Biol. 2023 Aug 15;23:394. doi: 10.1186/s12870-023-04410-9 (PMC10426221; doi:10.1186/s12870-023-04410-9)
Supplement: Supplementary file 2 — Additional file 2: Fig. S2. The effects of longitude, latitude, and altitude on the two principal component axes (T-PC1 and T-PC2) of the trait. Note: The solid line is significant and the dashed line is insignificant. [file 12870_2023_4410_MOESM2_ESM.docx]

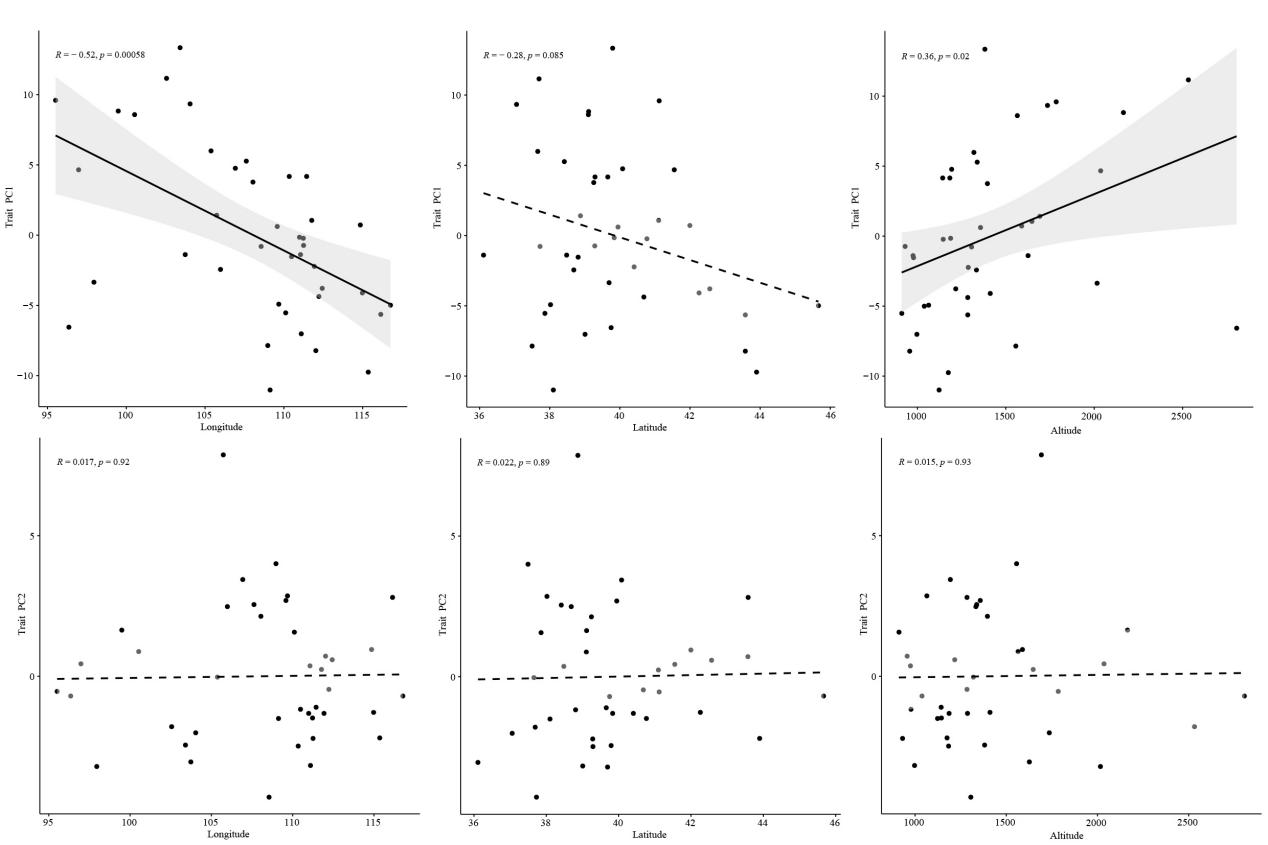


Fig.S2. The effects of longitude, latitude, and altitude on the two principal component axes (T-PC1 and T-PC2) of the trait. Note: The solid line is significant and the dashed line is insignificant.
