# Supplementary material for: Geographical variation in functional traits of leaves of Caryopteris mongholica and the role of climate
Source: BMC Plant Biol. 2023 Aug 15;23:394. doi: 10.1186/s12870-023-04410-9 (PMC10426221; doi:10.1186/s12870-023-04410-9)
Supplement: Supplementary file 3 — Additional file 3: Fig. S3. Response curves between the probability of presence and climate variables of C. mongholica. The y-axis is the probability of existence of C. mongholica. Blue: mean±one standard deviation. [file 12870_2023_4410_MOESM3_ESM.docx]

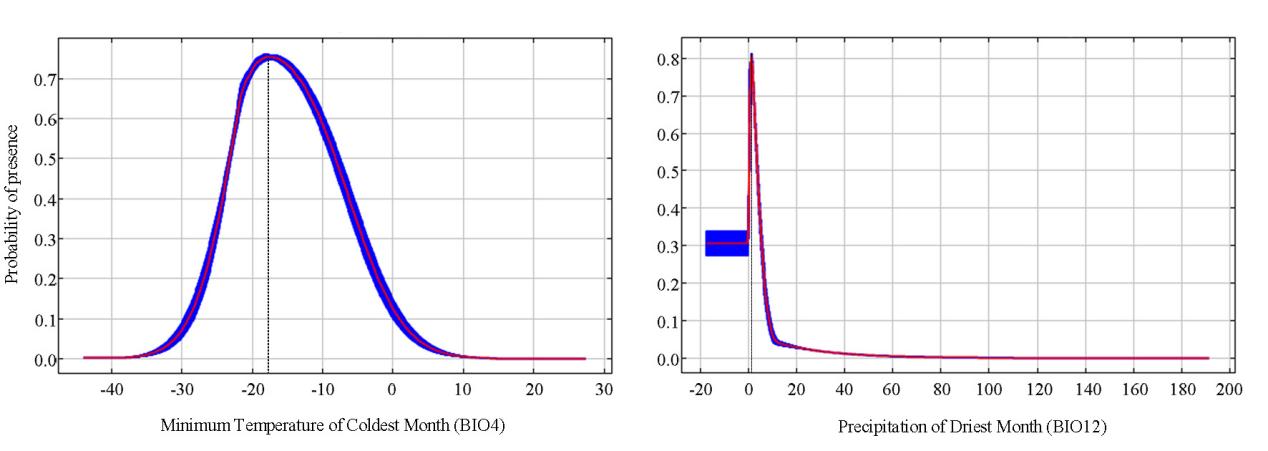


Fig.S3. Response curves between the probability of presence and climate variables of *C. mongholica*. The y-axis is the probability of existence of *C. mongholica*. Blue: mean±one standard deviation.
