# Supplementary material for: Geographical variation in functional traits of leaves of Caryopteris mongholica and the role of climate
Source: BMC Plant Biol. 2023 Aug 15;23:394. doi: 10.1186/s12870-023-04410-9 (PMC10426221; doi:10.1186/s12870-023-04410-9)
Supplement: Supplementary file 4 — Additional file 4: Table S1. Site characteristics for 40 sites of C. mongholica communities across its distribution in China. [file 12870_2023_4410_MOESM4_ESM.docx]

**Table** **S1.** Site characteristics for 40 sites of *C. mongholica* communities across its distribution in China.

| Site code | Location information | Latitude(N) | Longitude(E) | Altitude(m) |
| --- | --- | --- | --- | --- |
| 1 | Tumd Left Banner, Huhhot City,  Inner Mongolia Autonomous Region | 40°46′ | 111°13′ | 1123 |
| 2 | Wuchuan County, Huhhot City,  Inner Mongolia Autonomous Region | 41°06′ | 111°46′ | 1625 |
| 3 | Qingshuihe County, Huhhot City,  Inner Mongolia Autonomous Region | 39°39′ | 111°26′ | 1210 |
| 4 | Horinger County, Huhhot City,  Inner Mongolia Autonomous Region | 40°24′ | 111°55′ | 1299 |
| 5 | Dalad Banner, Ordos City,  Inner Mongolia Autonomous Region | 39°56′ | 109°34′ | 1349 |
| 6 | Wulan town, Otog Banner, Ordos City,  Inner Mongolia Autonomous Region | 39°15′ | 108°02′ | 1396 |
| 7 | Mengxi town, Otog Banner, Ordos City,  Inner Mongolia Autonomous Region | 40°04′ | 106°55′ | 1196 |
| 8 | Otog Front Banner, Ordos City,  Inner Mongolia Autonomous Region | 38°24′ | 107°36′ | 1326 |
| 9 | Uxin Banner, Ordos City,  Inner Mongolia Autonomous Region | 37°43′ | 108°33′ | 1321 |
| 10 | Jungar Banner, Ordos City,  Inner Mongolia Autonomous Region | 39°50′ | 110°58′ | 1271 |
| 11 | Liangcheng County, Ulanqab City,  Inner Mongolia Autonomous Region | 40°40′ | 112°13′ | 1336 |
| 12 | Sonid Right Banner, Xilingol League,  Inner Mongolia Autonomous Region | 42°33′ | 112°25′ | 1269 |
| 13 | Erenhot County, Xilingol League,  Inner Mongolia Autonomous Region | 43°34′ | 112°01′ | 971 |
| 14 | Abag Banner, Xilingol League,  Inner Mongolia Autonomous Region | 43°53′ | 115°21′ | 1190 |
| 15 | Dong Ujimgin Banner, Xilingol League,  Inner Mongolia Autonomous Region | 45°39′ | 116°45′ | 1018 |
| 16 | Xilinhot County, Xilingol League,  Inner Mongolia Autonomous Region | 43°34′ | 116°08′ | 1295 |
| 17 | Zhengxiangbai Banner, Xilingol League,  Inner Mongolia Autonomous Region | 42°15′ | 114°58′ | 1421 |
| 18 | Alxa Right Banner, Alxa League,  Inner Mongolia Autonomous Region | 39°47' | 103°24' | 1382 |
| 19 | Alxa Left Banner, Alxa League,  Inner Mongolia Autonomous Region | 38°52′ | 105°43′ | 1654 |
| 20 | Shapotou District, Zhongwei City,  Ningxia Hui Autonomous Region | 37°39′ | 105°22′ | 1332 |
| 21 | Helan County, Yinchuan City,  Ningxia Hui Autonomous Region | 38°41′ | 105°58′ | 1329 |
| 22 | Kangbao County, Zhangjiakou City,  Hebei Province | 41°59′ | 114°50′ | 1621 |
| 23 | Daliuta town, Shenmu County, Yulin City,  Shaanxi Province | 39°17′ | 110°19′ | 1206 |
| 24 | Xigou street, Shenmu County, Yulin City,  Shaanxi Province | 38°41′ | 110°28′ | 978 |
| 25 | Hongshiqiao town, Yuyang District,  Yulin City,Shaanxi Province | 38°06′ | 109°07′ | 1123 |
| 26 | Zhenchuan town, Yuyang District, Yulin City,Shaanxi Province | 37°51′ | 110°06′ | 911 |
| 27 | Hengshan District, Yulin City,  Shaanxi Province | 38°01′ | 109°40′ | 1064 |
| 28 | Jingbian County, Yulin City,  Shaanxi Province | 37°29′ | 108°58′ | 1556 |
| 29 | Xing County, Lvliang City,  Shanxi Province | 38°28′ | 111°03′ | 975 |
| 30 | Baode County, Xinzhou City,  Shanxi Province | 39°00′ | 111°05′ | 997 |
| 31 | Hequ County, Xinzhou City,  Shanxi Province | 39°16′ | 111°15′ | 931 |
| 32 | Anning District, Lanzhou City,  Gansu Province | 36°06′ | 103°44′ | 1624 |
| 33 | Jingtai County, Baiyin City,  Gansu Province | 37°03' | 104°02' | 1735 |
| 34 | Liangzhou District, Wuwei City,  Gansu Province | 37°41' | 102°33' | 2532 |
| 35 | Ganzhou District, Zhangye City,  Gansu Province | 39°06′ | 100°31′ | 1565 |
| 36 | Sunan Yugur Autonomous County,  Zhangye City, Gansu Province | 39°60' | 99°28' | 2165 |
| 37 | Wenshu Town, Jiayuguan City,  Gansu Province | 39°41' | 97°56' | 2016 |
| 38 | Guazhou County, Jiuquan City,  Gansu Province | 41°70' | 95°30' | 1784 |
| 39 | Yingzui mountain, Subei Mongolian Autonomous County, Jiuquan City, Gansu Province | 39°44' | 96°21' | 2805 |
| 40 | Mazong mountain, Subei Mongolian Autonomous County, Jiuquan City, Gansu Province | 41°33' | 96°57' | 2036 |
