# Supplementary material for: Geographical variation in functional traits of leaves of Caryopteris mongholica and the role of climate
Source: BMC Plant Biol. 2023 Aug 15;23:394. doi: 10.1186/s12870-023-04410-9 (PMC10426221; doi:10.1186/s12870-023-04410-9)
Supplement: Supplementary file 5 — Additional file 5: Table S2. Bivariate relationships among climatic variables. [file 12870_2023_4410_MOESM5_ESM.docx]

**Table S2.** Bivariate relationships among climatic variables.

| Variable code | Bio1 | Bio2 | Bio3 | Bio4 | Bio5 | Bio6 | Bio7 | Bio8 | Bio9 | Bio10 | Bio11 | Bio12 | Bio13 | Bio14 | Bio15 | Bio16 | Bio17 |
| --- | --- | --- | --- | --- | --- | --- | --- | --- | --- | --- | --- | --- | --- | --- | --- | --- | --- |
| Bio2 | -0.476** |  |  |  |  |  |  |  |  |  |  |  |  |  |  |  |  |
| Bio3 | 0.803** | 0.097 |  |  |  |  |  |  |  |  |  |  |  |  |  |  |  |
| Bio4 | 0.891** | -0.790** | 0.477** |  |  |  |  |  |  |  |  |  |  |  |  |  |  |
| Bio5 | -0.412** | 0.951** | 0.193 | -0.770** |  |  |  |  |  |  |  |  |  |  |  |  |  |
| Bio6 | 0.674** | 0.249 | 0.942** | 0.346* | 0.298 |  |  |  |  |  |  |  |  |  |  |  |  |
| Bio7 | 0.754** | -0.831** | 0.354* | 0.882** | -0.728** | 0.197 |  |  |  |  |  |  |  |  |  |  |  |
| Bio8 | 0.831** | 0.09 | 0.979** | 0.514** | 0.136 | 0.932** | 0.335* |  |  |  |  |  |  |  |  |  |  |
| Bio9 | 0.908** | -0.798** | 0.511** | 0.985** | -0.728** | 0.354* | 0.914** | 0.528** |  |  |  |  |  |  |  |  |  |
| Bio10 | 0.286 | 0.013 | 0.169 | 0.152 | -0.047 | 0.104 | -0.038 | 0.288 | 0.155 |  |  |  |  |  |  |  |  |
| Bio11 | 0.236 | 0.114 | 0.18 | 0.062 | 0.061 | 0.108 | -0.122 | 0.296 | 0.073 | 0.983** |  |  |  |  |  |  |  |
| Bio12 | -0.118 | 0.103 | -0.178 | -0.181 | 0.073 | -0.255 | -0.215 | -0.103 | -0.153 | 0.646** | 0.661** |  |  |  |  |  |  |
| Bio13 | -0.242 | 0.664** | 0.109 | -0.531** | 0.672** | 0.091 | -0.618** | 0.123 | -0.495** | 0.326* | 0.464** | 0.234 |  |  |  |  |  |
| Bio14 | 0.234 | 0.096 | 0.168 | 0.071 | 0.043 | 0.103 | -0.108 | 0.281 | 0.080 | 0.993** | 0.993** | 0.654** | 0.422** |  |  |  |  |
| Bio15 | -0.067 | 0.136 | -0.12 | -0.161 | 0.093 | -0.188 | -0.233 | -0.03 | -0.139 | 0.781** | 0.795** | 0.944** | 0.281 | 0.787** |  |  |  |
| Bio16 | 0.177 | 0.131 | 0.123 | 0.018 | 0.069 | 0.065 | -0.163 | 0.237 | 0.024 | 0.986** | 0.991** | 0.668** | 0.457** | 0.997** | 0.798** |  |  |
| Bio17 | -0.099 | 0.116 | -0.162 | -0.177 | 0.08 | -0.237 | -0.228 | -0.078 | -0.151 | 0.765** | 0.777** | 0.945** | 0.268 | 0.771** | 0.997** | 0.783** |  |
| **P*< 0.05, ***P*< 0.01. | | | | | | | | | | | | | | | | | |

***Appendix:* The climate codes are as follows**:

BIO1 = Annual Mean Temperature;

BIO2 = Temperature Seasonality (standard deviation *100);

BIO3 = Maximum Temperature of Warmest Month;

BIO4 = Minimum Temperature of Coldest Month;

BIO5 = Temperature Annual Range (BIO3-BIO4);

BIO6 = Mean Temperature of Wettest Quarter;

BIO7 = Mean Temperature of Driest Quarter;

BIO8 = Mean Temperature of Warmest Quarter;

BIO9 = Mean Temperature of Coldest Quarter;

BIO10 = Annual Precipitation;

BIO11 = Precipitation of Wettest Month;

BIO12 = Precipitation of Driest Month;

BIO13 = Precipitation Seasonality (Coefficient of Variation);

BIO14 = Precipitation of Wettest Quarter;

BIO15 = Precipitation of Driest Quarter;

BIO16 = Precipitation of Warmest Quarter;

BIO17 = Precipitation of Coldest Quarter.
