# Supplementary material for: Geographical variation in functional traits of leaves of Caryopteris mongholica and the role of climate
Source: BMC Plant Biol. 2023 Aug 15;23:394. doi: 10.1186/s12870-023-04410-9 (PMC10426221; doi:10.1186/s12870-023-04410-9)
Supplement: Supplementary file 6 — Additional file 6: Table S3. Principal components analysis for climatic variables estimated for provenances (40 sites) of C. mongholica. [file 12870_2023_4410_MOESM6_ESM.docx]

**Table S3.** Principal components analysis for climatic variables estimated for provenances (40 sites) of *C. mongholica*.

|  | PC1 | PC2 | Latitude | Longitude | Altitude |
| --- | --- | --- | --- | --- | --- |
| Eigenvalue | 3.9 | 1.9 |  |  |  |
| Percentage variation explained | 49.241 | 24.251 |  |  |  |
| Eigenvectors |  |  |  |  |  |
| BIO1 = Annual Mean Temperature | 0.863 | 0.441 | -0.751** | -0.121 | -0.418** |
| BIO2 = Temperature Seasonality (standard deviation*100) | -0.806 | 0.255 | 0.885** | 0.716** | -0.541** |
| BIO3 = Maximum Temperature of Warmest Month | 0.476 | 0.545 | -0.244 | 0.182 | -0.761** |
| BIO4 = Minimum Temperature of Coldest Month | 0.972 | 0.126 | -0.907** | -0.405** | -0.009 |
| BIO7 = Mean Temperature of Driest Quarter | 0.950 | -0.075 | -0.848** | -0.644** | 0.195 |
| BIO10 = Annual Precipitation | 0.017 | 0.850 | -0.293 | 0.607** | -0.515** |
| BIO12 = Precipitation of Driest Month | -0.268 | 0.583 | 0.022 | 0.391* | -0.166 |
| BIO13 = Precipitation Seasonality (Coefficient of Variation) | -0.631 | 0.547 | 0.480** | 0.690** | -0.576** |
| PC1 |  |  | -0.903** | -0.705** | 0.499** |
| PC2 |  |  | 0.014 | 0.810** | -0.679** |
| **P*< 0.05, ***P*< 0.01 | | | | |  |
